# *Bacillus cereus* strain S2 shows high nematicidal activity against *Meloidogyne incognita* by producing sphingosine

**DOI:** 10.1038/srep28756

**Published:** 2016-06-24

**Authors:** Huijuan Gao, Gaofu Qi, Rong Yin, Hongchun Zhang, Chenggang Li, Xiuyun Zhao

**Affiliations:** 1 Huazhong Agricultural University, College of Life Science and Technology, Wuhan, 430070, PR China

## Abstract

Plant-parasitic nematodes cause serious crop losses worldwidely. This study intended to discover the antagonistic mechanism of *Bacillus cereus* strain S2 against *Meloidogyne incognita*. Treatment with *B. cereus* strain S2 resulted in a mortality of 77.89% to *Caenorhabditis elegans* (a model organism) and 90.96% to *M. incognita*. In pot experiment, control efficiency of *B. cereus* S2 culture or supernatants were 81.36% and 67.42% towards *M. incognita*, respectively. In field experiment, control efficiency was 58.97% towards *M. incognita*. Nematicidal substances were isolated from culture supernatant of *B. cereus* S2 by polarity gradient extraction, silica gel column chromatography and HPLC. Two nematicidal compounds were identified as C16 sphingosine and phytosphingosine by LC-MS. The median lethal concentration of sphingosine was determined as 0.64 μg/ml. Sphingosine could obviously inhibit reproduction of *C. elegans*, with an inhibition rate of 42.72% for 24 h. After treatment with sphingosine, ROS was induced in intestinal tract, and genital area disappeared in nematode. Furthermore, *B. cereus* S2 could induce systemic resistance in tomato, and enhance activity of defense-related enzymes for biocontrol of *M. incognita*. This study demonstrates the nematicidal activity of *B. cereus* and its product sphingosine, as well provides a possibility for biocontrol of *M. incognita*.

Root-knot nematodes *Meloidogyne* spp. are worldwidely distributed plant parasites. These nematodes can parasitize to cause serious damages to many important agricultural crops such as potato, cotton, tomato and etc^1^. After being infected, the plant roots are damaged to loss their functions to normally absorb water and nutrients from soils. The most damaging *Meloidogyne* species are *M. arenaria*, *M. incognita*, *M. javanica* and *M. hapla*^2^,^3^. The global annual loss of crops is about multiple billions of dollars due to *M. incognita* infection.

During the past several decades, many studies are tried to control nematode by different strategies including rotation of non-host crops, application of nematicidal chemicals such as halogenated aliphatic hydrocarbons (e.g., 1,3-dichloropropene), methyl isothiocynate mixtures, oxamyl, Thionazin and carbofuran^4^,^5^,^6^. However, these chemical nematicides are highly toxic compounds, which can cause severe harm to environment after being abused in soils^7^,^8^. Thereby, novel nematicides with higher safety and efficiency are necessary for protecting crops from nematode infestation. Instead the biocontrol agents such as *Bacillus* spp. have drawn attentions in recent years because of their safety to environment, humans and animals^9^.

Various nematode-pathogen bacteria including *Pasteuria*, *Pseudomonas* and *Bacillus*, have been isolated from soil, host-plant and nematodes for biocontrol of the plant-parasitic nematodes^9^,^10^,^11^,^12^. These bacteria kill nematodes by different mechanisms, including parasitizing and interfering nematodes to recognize plants, producing anti-nematode toxins and enzymes, and so on. These bacteria also play their anti-nematode activity by promotion of plant growth and improvement of plant resistance against nematodes^10^,^13^.

*Bacillus* spp. can synthesize various molecules that are toxic to nematodes^11^,^14^,^15^,^16^. For example, *B. thuringiensis* shows nematicidal activity towards *M. incognita* and *Helerodera glycines* by producing crystal inclusions, a family of toxic proteins to a wide range of insect species including nematode^17^,^18^,^19^. However, there is no report about the nematicidal activity of *B. cereus*. *B. cereus* is a potential resource for biocontrol of pests. For example, a strain of *B. cereus* isolated from *Myrmeleon bore* can secrete sphingomyelinase C to paralyze and kill *German cockroaches* and *Blattela germanica*^20^. Six *B. cereus* strains are reported to produce several small, nonproteinaceous insecticidal exotoxins^21^,^22^,^23^. A novel mosquitocidal *Bacillus cereus* VCRC-B520 isolated from marine soil can produce an endotoxin-specific insecticidal protein of Cry4Aa^24^.

We previously isolated a *Bacillus cereus* strain S2 with high nematicidal activity against *M. incognita*. In this study, the anti-nematode mechanism was investigated for *B. cereus* S2, and found this strain could produce sphingosine to induce ROS in *M. incognita*. Accumulation of ROS destroyed the genital area to inhibit nematode reproduction. Moreover, this strain could also induce systemic plant resistance against *M. incognita*.

## Results

### *B. cereus* strain S2 producing extracellular substances for nematicidal activity

**Figure 1:**
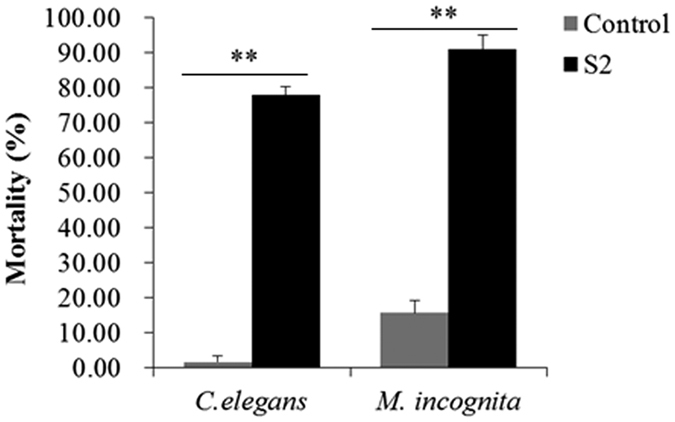
Detecting effects of *B*. *cereus* S2 supernatant on *C.elegans* and *M*. incognita. Control: M9 buffer. **indicated very significant (P < 0.01) difference between two groups.

*B. cereus* strain S2 showed high nematicidal activity (Fig. 1). Treatment with the supernatant of *B. cereus* strain S2 culture resulted in a mortality of 77.89% to *Caenorhabditis elegans* (a model organism) and 90.96% to *M. incognita*, respectively. The results indicated that *B. cereus* strain S2 can produce some extracellular substances to kill nematodes.

### Characterization of nematicidal activity of *B. cereus* strain S2

The nematicidal substances of *B. cereus* strain S2 were analyzed for their stability at different pH values. It was found the supernatant of *B. cereus* strain S2 culture retained with the high nematicidal activity at a broad pH range from 2.0 to 8.0. However, this nematicidal activity obviously reduced at a pH value more than 10.0 (Fig. S1). This result indicated the nematicidal substances of *B. cereus* strain S2 are stable in acidic, neutral and weak basic enviroments, but not stable in strong basic enviroments.

The nematicidal substances of *B. cereus* strain S2 were also analyzed for their stability towards protease digestion. After hydrolysis with protease, the supernatant of *B. cereus* strain S2 culture still showed a high nematicidal activity with a mortality of 85.9% towards *C. elegans*, similar to the supernatant without treatment by protease (Fig. S1). This result suggested that the nematicidal substances of *B. cereus* strain S2 are not proteins but possibly some secondary metabolites. Thereby, we firstly isolated the nematicidal substances by organic solvent extraction in the following studies.

### Identifying nematicidal substances as sphinganine and phytosphingosine

**Figure 2:**
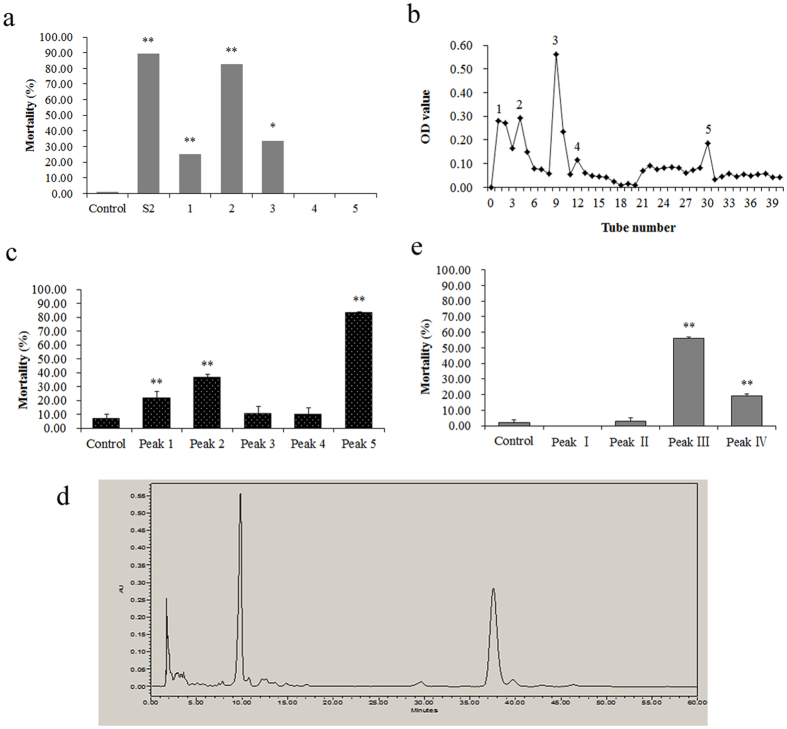
Purification and identification of nematicidal substance from *B*. *cereus* S2. (**a**) *B. cereus* culture was extracted with different organic solvent, then used for detecting the nematicidal activity on *C. elegans*. S2, *B. cereus* S2 culture; 1–4, extracted phase of petroleum ether, chloroform, ethyl acetate and n-butyl alcohol, respectively; 5, water phase. (**b**) Five peaks were separated by silica gel column chromatography. 1–5, Peak 1 to 5. (**c**) Nematicidal activity of five peaks separated by silica gel column chromatography on *C. elegans*. (**d**) Four elution peaks were collected from HPLC. 1–4, peak I–IV. (**e**) Nematicidal activity of four peaks purified by HPLC on *C. elegans*. *indicate the treatment groups are significantly (*P* < 0.05) different from the control. **indicate the treatment groups are very significantly (*P* < 0.01) different from the control.

The supernatant of *B. cereus* strain S2 culture was extracted with petroleum ether, chloroform, ethyl acetate or n-butyl alcohol, respectively. The extracted phase by chloroform showed the highest nematicidal activity with a mortality of 82.51% towards *C. elegans*, similar to the *B. cereus* S2 culture with a mortality of 89.59% (Fig. 2). Besides, the nematicidal substances could also be partly extracted by ethyl acetate and petroleum ether, but not be extracted by n-butyl alcohol. These results indicated that the nematicidal substances are with weak polarity and maybe non-polar molecules. Thereby, we further purified the nematicidal substances with silica gel column chromatography.

After being extracted by chloroform, the nematicidal substances were isolated through a silica gel column. By elution with CH_2_Cl_2_-MeOH, five peaks were collected for determining the nematicidal activity (Fig. 2). Peak 5 showed the highest nematicidal activity with a mortality of 83.24% towards *C. elegans*, significantly (*P *< 0.01) higher than the control and other peaks (Fig. 2). Peak 2 and Peak 1 also showed moderate nematicidal activity with a mortality of 36.53% and 21.67% towards *C. elegans*, respectively.

The highest nematicidal activity was found in Peak 5, so the substances in Peak 5 were further purified by HPLC. By elution with acetonitrile-water, total 4 elution peaks were collected for determining the nematicidal activity (Fig. 2). The substances in Peak III showed the highest nematicidal activity with a mortality of 56.19% towards *C. elegans* (Fig. 2). Besides, the substance in Peak IV also showed a weak nematicidal activity with a mortality of 19.29% towards *C. elegans*.

**Figure 3:**
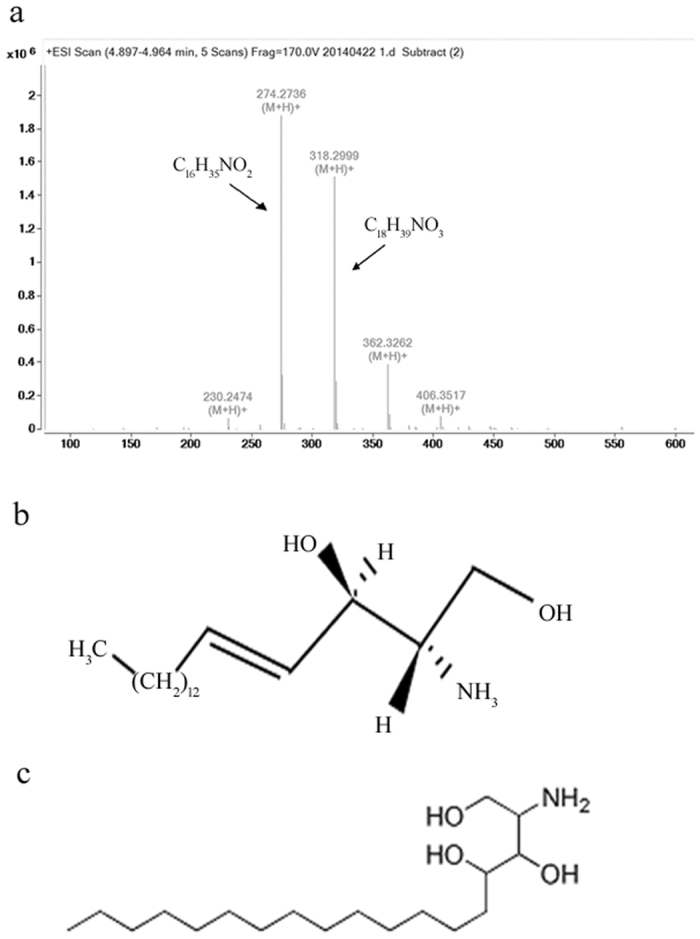
Nematicidal substances analyzed by LC-MS. (**a**) The positive ion ESI-m (m/z) of compound 1 and compound 2; (**b**) Molecular formula of compound 1; (**c**) Molecular formula of compound 2.

The nematicidal substances in Peak III were analyzed with LC-MS (Fig. 3). The results showed that Peak III contained two substances. The positive ion ESI-m (m/z) of substance 1 and substance 2 were 274.3 Da and 318.3 Da, respectively. These two substances were retrieved in meplin data base and compared with standard spectrum, and found they belonged to the sphingosines family. Substance 1 was identified as C16 sphinganine with a deduced molecular formula of C_16_H_35_NO_2_ (Fig. 3). Substance 2 was identified as C18 phytosphingosine with a deduced molecular formula of C_18_H_39_NO_3_ (Fig. 3).

### Sphingosine with a lethal action towards *C. elegans*

**Figure 4:**
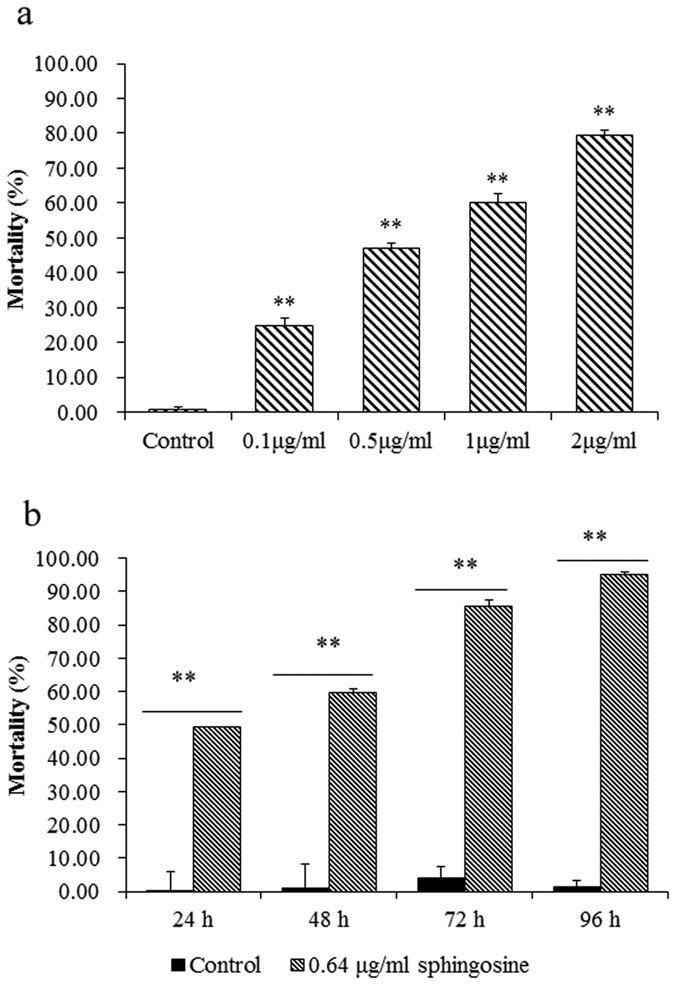
Nematicidal activity of sphingosine on *C*. *elegans*. (**a**) Nematicidal activity of different concentrations of sphingosine on *C. elegans*; (**b**) Nematicidal activity of sphingosine on *C. elegans* for different times (24, 48, 72 and 96 h). **indicated very significant (*P* < 0.01) difference between sphingosine treatment and control.

The nematicidal activities were detected for the different concentrations of sphingosine (Fig. 4). The mortality of 0.1, 0.5, 1 and 2 μg/ml of sphingosine was 24.8%, 46.99%, 60.09% and 79.35%, respectively. The median lethal concentration (LC_50_) of sphingosine was determined as 0.64 μg/ml by analysis with SPASS software. This result showed that sphingosine is very efficient for killing nematodes, which is the main nematicidal substance produced by *B. cereus* strain S2.

The nematicidal activities were also detected after treatment by sphingosine for different times (Fig. 4). After treatment with sphingosine (0.64 μg/ml) for 24, 48, 72 and 96 h, the mortality was determined as 49.53%, 59.64%, 85.67% and 95.07% towards *C. elegans*, respectively. Along with the treatment time, the mortality of sphingosine towards *C. elegans* obviously increased, indicating the lethal action of sphingosine is with an obvious time effects.

### Sphingosine inducing ROS in nematode

**Figure 5:**
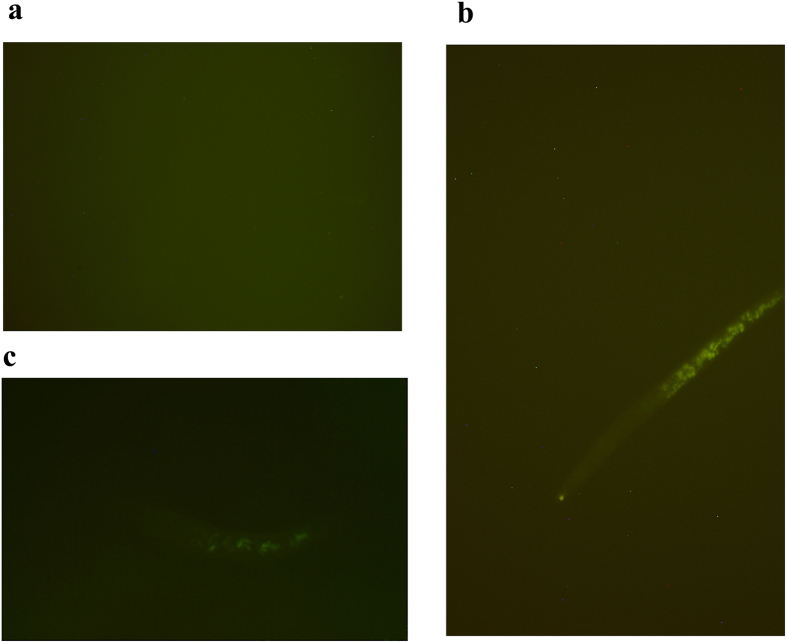
ROS induced in *C*. *elegans* by sphingosine. (**a**) *C. elegans* treated with M9 buffer as control; (**b**) *C. elegans* treated with sphingosine (0.64 μg/ml) was observed under a fluorescence microscope.

After treated with sphingosine, a robust of reactive oxygen (ROS) was found in the intestinal tract of nematode (Fig. 5), while not in the control nematodes. ROS can induce oxidative injury, cell apoptosis and cell necrosis *in vitro* and *in vivo*^25^,^26^. Thereby, the nematicidal activity of sphingosine may be attributed to its ability to induce ROS in nematodes.

### Sphingosine destroyed genital areas for inhibiting nematode reproduction

**Figure 6:**
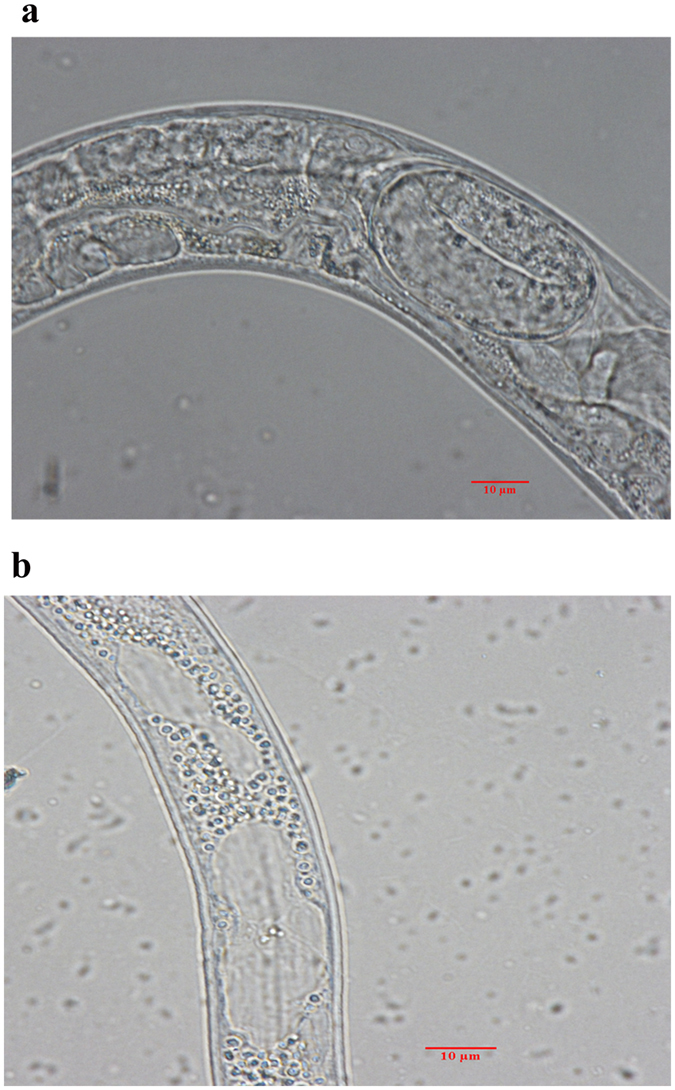
Structures change of *C*. *elegans* induced by sphingosine. (**a**) *C. elegans* treated with M9 buffer as control; (**b**) *C. elegans* treated with sphingosine (0.64 μg/ml) was observed under a differential interference contrast microscope.

After treatment with sphingosine, the internal structures of nematode were observed by a differential interference contrast microscope (Fig. 6). The results showed many bubbles were observed in the nematodes treated with sphingosine, while not in the control. Furthermore, the genital areas could not be observed in the sphingosine-treated nematodes. This result clearly showed that sphingosine can act on the reproductive area and destroy the internal structure of nematode, therefore make lethal effect on nematode as well suppress nematode reproduction.

**Figure 7:**
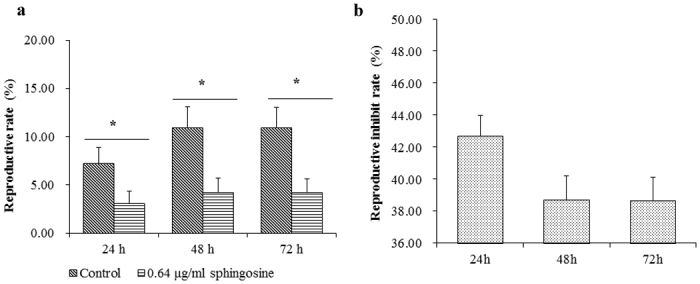
Sphingosine inhibiting reproduction of *C*. *elegans*. (**a**) Reproductive rate of *C. elegans* treated by sphingosine (0.64 μg/ml) at different time points; (**b**) Inhibitory rate of sphingosine on *C. elegans* reproduction at different time points.

As described above, sphingosine could destroy the genital areas of nematode. Thereby, we further determined its effects on nematode reproduction. The results showed sphingosine could significantly inhibit nematode reproduction. After treatment with sphingosine (0.64 μg/ml ) for 24, 48 and 72 h, the reproduction of nematode decreased by 42.72%, 38.72% and 38.66%, respectively. The number of nematode offspring in sphingosine-treated group was significantly (*P *< 0.05) lower than that in the control group (Fig. 7). This result indicated that sphingosine can inhibit nematode reproduction and fertility.

### *B. cereus* strain S2 showing excellent biocontrol effects on *M. incognita*

In the pot experiment, *M. incognita* could infect and form lots of large root galls in the control group, while only less and smaller galls were formed in the tomato root after treatment with *B. cereus* strain S2. Disease index in the control group was 34.07, while it was 6.35 and 11.1 in the *B. cereus* S2 culture group and the *B. cereus* S2 supernatant group, respectively (Table 1, Fig. S2). Both *B. cereus* S2 culture and supernatant showed an excellent control effect on the root-knot nematode. The control efficiency towards *M. incognita* was 67.42% for the *B. cereus* S2 supernatant, due to the key nematicidal substance of sphingosine in the supernatant. Comparision with the supernatant, the *B. cereus* S2 culture showed a higher control effect on *M. incognita* with a control efficiency of 81.36%. This can be explained that the *B. cereus* S2 culture contains not only sphingosine but also live cells or spores, which can colonize in the rhizosphere soil of tomato to exert nematicidal activity for a long term.

**Figure 8:**
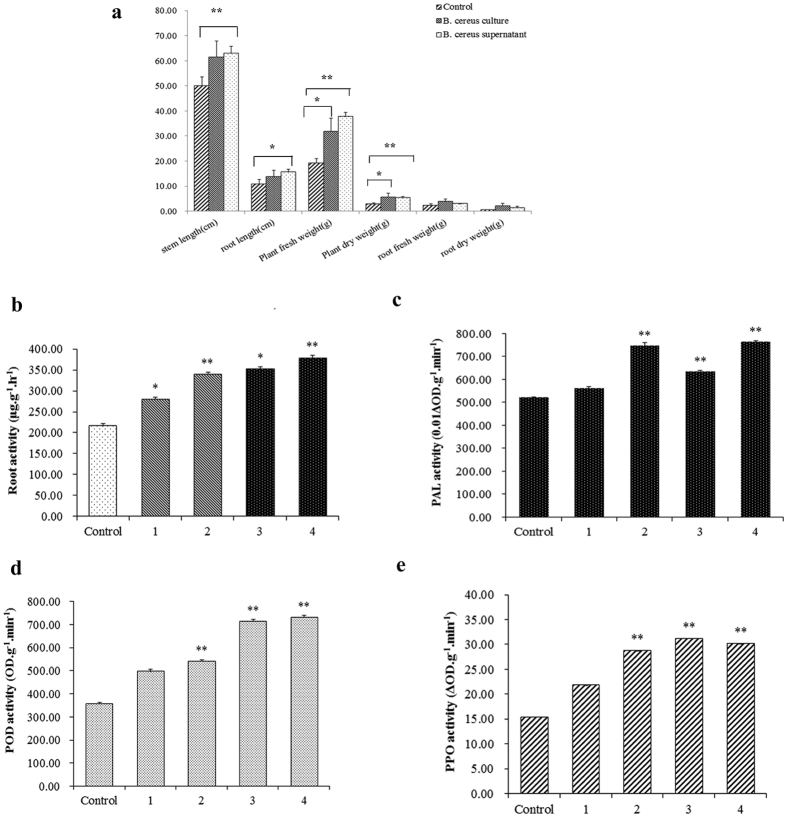
Defense-related enzymes and root activity in tomato treated by *B. cereus* S2. (**a**) Growth characteristics of tomato; (**b**) Root activity of tomato; (**c**) PAL activity of tomato; (**d**) POD activity of tomato; (**e**) PPO activity of tomato. Control, irrigating with water. 1, 2, Tomato inoculating with *B. cereus* S2 supernatant at 1 or 0 grade disease. 3, 4, Tomato inoculating with *B. cereus* S2 culture at 1 or 0 grade disease. *indicate significant (*P* < 0.05) difference between two groups. **indicate very significant (*P* < 0.01) difference between two groups.

Besides effectively control root-knot nematode disease, *B. cereus* S2 could also improve tomato growth (Fig. 8, Table 2). After irrigating with *B. cereus* strain S2 culture, the plant height, root length and plant dry weight of tomato seedlings respectively increased by 22.84%, 27.16% and 94.63%, accordingly when compared with the control. The plant dry weight was significantly (*P *< 0.05) higher than the control. Additionally, the plant height, root length and plant dry weight also increased by 25.79%, 44.29% and 82.11% after irrigating with *B. cereus* strain S2 supernatant. Analysis by SPSS, it was found the plant height and plant dry weight were both significantly (*P *< 0.01) higher than the control.

Treatment with *B. cereus* S2 culture or supernatant could also improve the root activity of tomato. The root activity increased by 57.21% and 48.64% than the control after irrigating with *B. cereus* strain S2 culture or supernatant, respectively (Fig. 8). The root activity of tomato inoculating with *B. cereus* S2 culture or supernatant were both significantly (*P *< 0.05) higher than the control. These results indicated that inoculating with *B. cereus* strain S2 could improve the root activity of tomato seedlings for better nematicidal effects.

Treatment with *B. cereus* strain S2 could also increase the activities of defense-related enzymes. The activities of POD, PPO and PAL were induced in tomato seedlings after inoculating with *B. cereus* strain S2 (Fig. 8). The PAL, POD and PPO activity respectively increased by 43.8%, 51.8% and 86.2% in the tomato seedlings treated with *B. cereus* strain S2 culture when compared to the control. The activities of three defense-related enzymes were all significantly higher (*P *< 0.01) than the control. Thereby, *B. cereus* can also activate the plant defense systems for control of *M. incognita*.

In the field experiment, the biocontrol efficiency on *M. incognita* was 58.97% for the *B. cereus* strain S2 culture. The disease index of *B. cereus* strain S2 culture was very significantly (*P *< 0.01) lower than the control. This result showed the *B. cereus* strain S2 culture is very effective for biocontrol of root-knot nematode in the fields. But the nematicidal effect of *B. cereus* strain S2 was lower than the nematicide Avermectin with a control efficiency of 73.19% in this study (Table 3).

## Discussion

Root-knot nematodes are important plant pathogens, which can cause severe damages to crops worldwildly. The frequently used nematicides are chemical nematicides. It was known that the chemical nematicides have deleterious effects on environment and human health, so researchers tried to find antagonistic microorganisms for biocontrol of nematodes in decades. Many fungi and bacteria were reported with nematicidal acitivities by producing the nematicidal secondary metabolites. For examples, some rhizosphere bacteria such as *Pseudomonas fluorescens*, *P. aeruginosa*, *Bacillus thuringiensis* and *B. firmus* are reported with nematicidal activity^16^,^18^,^27^, by producing nematicidal substances (etc. parasporal crystal, β-exotoxin), competing spatial sites and nutritional sites with root-knot nematodes, changing the action mode between root exudates and nematodes^10^,^28^,^29^,^30^,^31^,^32^,^33^. During the periods of eggs hatching and infection, nematodes were very susceptible to root exudates. After interaction with plant roots, rhizosphere bacteria can change the root exudates to indirectly interfere with the infection of roots by nematodes^10^,^28^. Several proteins are extensively studied as nematicidal substances including chitinase, β-exotoxin, α-exotoxin and δ-exotoxin^29^. These toxins can suppress nematode reproduction and egg hatching^10^. Some secondary metabolites of small molecule substances could also kill *C. elegans*, such as linoleic acid, citric acid, oxalic acid, 2,4-diacetylphloroglucinol and hydrogen sulphide etc^30^. For example, *Coronophora gregaria* produces an aliphatic compound MK7924 to kill *C. elegans*^31^. *Pseudomonas fluorescens* kills soybean cyst nematode by producing 2,4-diacetylphloroglucinolc^32^. *Corynebacterium paurometabolu* inhibits nematode egg hatching by producing hydrogen sulphide^33^.

We previously isolated five nematicidal bacteria strains from the rhizosphere soils of tomato. Among them, *B. cereus* strain S2 could produce nematicidal substances, which were stable at high temperatures and acidic conditions. These nematicidal substances were purified by extraction, silica gel column chromatography and HPLC. After analysis by LC-MS, the nematicidal substances were identified as sphingosine. This was the first report that *B. cereus* could produce nematicidal sphingosine. Sphingosine is a kind of lipids, which can be used as an anti-inflammatory agent and an antiseptics^34^. Sphingosine is safety for enviroment, human and animals, but very toxic to nematodes with a nematicidal LC_50_ value of 0.64 μg/ml. Therefore, sphingosine is a safe and effective nematicidal agent. After treatment by sphingosine, ROS was induced in nematode. This result indicated that sphingosine can induce ROS following with oxygen injury *in vivo*, therefore lead lethal action to nematode. ROS is also an important cellular message for inducing cell apoptosis and necrosis^25^,^26^, so the accumulated ROS may act as a message to induce cell apoptosis and necrosis in nematodes. As a subsequent result of ROS accumulation, 95.07% of nematodes were killed after treatment by sphingosine for 96 h. Many bubbles appeared, and the genital areas disappeared in the nematode body.

Sphingosine can severely inhibit nematode reproduction. This may be explained by the characteristics of sphingosine. Sphingosine is an 18-carbon amino alcohol with an unsaturated hydrocarbon chain. Thereby, sphingosine can easily act with the tissues or organs enriched with lipids. The reproduction organ and eggs are just the tissues enriched with lipids, thereby easily susceptible for the action of sphingosine. Otherwise, sphingosine can be phosphorylated to sphingosine-1-phosphate *in vivo*, which is an intracellular mediator to regulate cell survival^35^,^36^,^37^. Thereby, sphingosine possibly acts as a message to induce cell death in nematodes.

After irrigating with *B. cereus* strain S2 culture, the tomato height, weight, and root length were obviously higher than the control. It indicated that *B. cereus* strain S2 can improve tomato growth. The disease index of control group was 34.07, significantly higher than the group treated with *B. cereus* strain S2. The biocontrol efficiency was 81.36% and 67.42% for the *B. cereus* S2 culture and supernatant, respectively. The root activity of tomato treated with *B. cereus* strain S2 was also much higher than the control. Systemic resistance can be induced by some rhizosphere bacteria. After interaction with plant, rhizosphere bacteria can obviously improve the plant systemic resistance against pathogens^38^. In this study, after inoculation with *B. cereus* strain S2, the activities of PPO, POD and PAL were obviously improved in tomato. PPO, POD and PAL were important defense enzymes of plants, which are positively correlated with the plant systemic resistance against pathogens^39^. Collectively, *B. cereus* strain S2 can improve tomato growth and enhance plant root resistance against nematode infection.

In a conclusion, *B. cereus* strain S2 is with a good biocontrol effect on root-knot nematode. *B. cereus* strain S2 can produce sphingosine to induce ROS accumulation and destroy the genital areas in nematodes, as well inhibit the nematode reproduction. Application of *B. cereus* strain S2 culture can improve plant growth and enhance tomato resistance against nematode. *B. cereus* strain S2 and its product sphingosine is potential for biocontrol of nematodes in crops and vegetables.

## Methods

### Bacteria and plant materials

*B. cereus* strain S2 was isolated from the soil of tomato field in Huazhong Agricultural University (Wuhan city, China) and stored at our laboratory. The tomato seeds were purchased from Qingfeng Seed Technology Co., ltd (Wuhan, China). Tomato seedlings were grown in a mixture of soil and sand (1:1) at 25 ± 2 °C and 16 h light period in greenhouse.

### Feeding of nematodes

*Caenorhabditis elegans* wild strain N2 was used as a nematode model organism. *C. elegans* strain N2 was grown on NGM agar plates (Peptone 2.5 g/L, NaCl 3 g/L, agar 17 g/L, K_2_HPO_4_-KH_2_PO_4_ 25 mM, MgSO_4_ 1 mM, cholesterol 0.05 mg/L, CaCl_2_ 1 mM) by feeding with *Escherichia coli* strain OP50 at 20 °C. The synchronized first-stage juveniles (L1) were used in the nematicidal bioassays.

*M. incognita* was maintained on the roots of tomato. Lots of galls formed in the tomato roots after infected with *M. incognita*. Nematode eggs were isolated from galls. Before detecting the nematicidal activity of bacteria, the galls were peeled off from root and placed in water at 20 °C until the second-stage juveniles (J2) were hatched. J2 juveniles were used in the following nematicidal bioassays.

### Detecting nematicidal activity of *B. cereus* strain S2

*B. cereus* strain S2 was incubated in lysogeny broth (LB) medium (10 g NaCl, 10 g tryptone, 5 g yeast extract, pH 7.2, 1000 ml H_2_O) at 28 °C and 200 rpm for 72 h. Then, the *B. cereus* S2 culture was centrifuged at 8000 rpm for 10 min. Supernatant was collected and used for nematicidal bioassay. 200 μl of supernatant and 30 to 40 numbers of *C. elegans* L1 juveniles were added to each well of 96-well plates. 100 μg/ml streptomycin and 100 μg/ml chloramphenicol were also added in each well to inhibit bacterial contamination. The nematodes were incubated at 20 °C for 48 h. After incubation, the mortality of nematode was counted. The nematodes without detectable movement were judged as dead. Tests were done with three replicates. LB medium was added in the assay as a control.

### Detecting stability of nematicidal activity of *B. cereus* strain S2 culture

The supernatant of *B. cereus* strain S2 fermentation was collected by centrifugation, then treated with 20 mg/ml of trypsin or proteinase K at 37 °C for 30 min. Thereafter, the nematicidal activity of supernatant was detected as described above. On the other hand, the supernatant was boiled at 100 °C for 5 min, then used for detecting the nematicidal activity. The supernatant of *B. cereus* strain S2 culture was also adjusted to a pH value from 2.0 to 12.0 by 0.5 M HCl or NaOH solution for 2 h incubation, then the nematicidal activity of supernatant was detected after the pH value was re-adjusted to 7.4.

### Purifying nematicidal substances from *B. cereus* strain S2 culture

The supernatant of *B. cereus* strain S2 culture was extracted with an equal volume of petroleum ether, chloroform, ethyl acetate or n-butyl alcohol, respectively. The extracted organic phases and aqueous phases were separately collected, evaporated at 55 °C until dry and then dissolved with distilled water. Nematicidal activity of the extracted substances was detected as described above. The extracted phase of chloroform showed the highest nematicidal activity and was used for the further purification.

The extracted fractions by chloroform were loaded to a silica gel column, then eluted with CH_2_Cl_2_-MeOH (8:2, *V/V*). Five subfractions were collected. Each subfraction was evaporated at 55 °C to dry, then dissolved in distilled water for the nematicidal activity assay as described above. Subfraction 5 showed the highest activity.

Subfraction 5 was dissolved in methanol and then loaded to Agela Technologies C18 column (250 × 4.6 mm id, 5 μm, 100 Å) of high-performance liquid chromatography (HPLC, Waters system). The nematicidal substances were eluted with acetonitrile: ultra-pure water (5:5, *V/V*) at 1 ml/min flow rate. Recorder was set at 200 nm. Four peaks were collected. Nematicidal activity of four peaks were tested, and found the third peak had the highest nematicidal activity.

The third peak was analyzed by LC-MS (Aglient Technologies 6540UHD Accurate-Mass Q-TOF LC-MS). The gas temperature was 350 °C. The gas flow rate was 9.0 L/min, atomizer pressure was 0.2756 MPa. The detection range was between 100 m/z and 1700 m/z. The molecular weight of nematicidal substances was detected. Chemical formula of compounds were retrieved and compared with the standard compounds in Meplin database. Two substances with nematicidal activity were identified as sphingosine and phytosphingosine, respectively.

### Detecting nematicidal activity of sphingosine

Sphingosine and phytosphingosine are similar chemical compounds belonging to sphingolipids family. We deduced these two compounds are with similar activities. Therefore, only the commercial sphingosine (99% purity, Xi’An Herbking Biotechnology Co., Ltd, China) was used for the further studies. The nematicidal activity of different concentrations of sphingosine (0.1, 0.5, 1, and 2 μg/ml, respectively) was tested against *C. elegans* by the methods described above for caculating the LC_50_ value of sphingosine. Each concentration of sphingosine was detected with three replicates.

### Detecting ROS production in *C. elegans* induced by sphingosine

Possible reactive oxygen species (ROS) induced by sphingosine was detected in nematode by fluorescent staining^40^. *C. elegans* was treated with 0.64 μg/ml of sphingosine for 48 h, then 250 μl fluorescence probe DCFH-DA (Sigma-Aldrich, USA) was added to each well and incubated at 37 °C for 30 min in dark. After staining, nematodes were washed with M9 buffer for three times, then ROS in nematode was observed under a fluorescence microscope. Nematode only treated with M9 buffer was used as a control.

### Analyzing reproductive rate of nematode treated by sphingosine

In order to know whether sphingosine could inhibit nematode reproduction, the four-stage juveniles of *C. elegans* were treated with 0.64 μg/ml sphingosine for 12, 24, 48 and 72 h, then the number of nematodes and eggs were counted, respectively^41^. *C. elegans* only treated with M9 buffer was used as a control.

### Determining control efficiency of *B. cereus* strain S2 on *M. incognita* by pot experiment

In the pot experiment, each plastic pot was filled with soil mixtures (sand and organic matter, 1:1). Each four-leaves stage tomato seedling was transplanted into one pot and cultivated in greenhouse at 25 ± 2 °C. About 2000 J2 juveniles of *M. incognita* were inoculated to the rhizosphere soils of each seedling. *B. cereus* strain S2 was grown in LB medium at 28 °C for 72 h by shaking at 200 rpm. Post-transplantation for 20, 40 and 60 d, each tomato seedling was irrigated with 25 ml of *B. cereus* S2 culture, supernatant or just water (negative control) around the roots. Three replicates were set up for each treatment, and 15 tomato seedlings were treated in each replicate. Post-transplantation for 90 d, the severity of root galling was assessed^42^. Disease index (DI) and control efficiency were calculated according to the formulas:


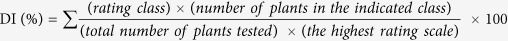



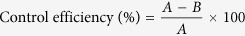


where A and B are DI in the control and test treatments, respectively.

The root activity of three treatments was detected through following procedures. 0.5 g tomato roots were placed in 10 ml mixtures of 0.4% 2,3,5-Triphenyltetrazolium chloride (TTC) solution and phosphate buffer (1:1, *V/V*), and incubated at 37 °C for 2 h in dark. After incubation, 10 ml of ethyl acetate was added for thoroughly homogenizing the roots. The quantity of reduced four azole nitrogen was determined at 485 nm by a spectrophotometer for caculating the activity of roots^43^. Additionally, the shoot high, root length, shoot fresh weight, shoot dry weight, root fresh weight and dry weight of each seeding was also measured in this study.

### Determining defense-related enzymes activity induced by *B. cereus* strain S2

Totamto seedlings cultured in pots were treated by *B. cereus* S2 culture, supernatant or just water as described above, then the activity of defense-related enzymes including phenylalanine ammonia lyase (PAL), polyphenol oxidase (PPO) and peroxidase (POD) were detected in seedlings following the procedures of González-Aguilar^44^, Flurkey^45^ and Rathmell^46^, respectively.

### Determining control efficiency of *B. cereus* strain S2 on *M. incognita* by field experiment

In the field experiment, tomato seedlings were planted into the soils infected with *M. incognita* during the former years at Enshi, Hubei province (China). *B. cereus* strain S2 was grown in LB medium at 28 °C for 72 h by shaking at 200 rpm. Post - plantation for 10, 30 and 60 d, each tomato seedling was irrigated with 200 ml of *B. cereus* strain S2 culture (10^9^ CFU/ml), 0.05% Avermectin pesticide (positive control) or just water (negative control) around the roots. Three plots were set up in each treatment, and 60 tomato seedlings were planted in each plot. Plots were randomly arranged. Post - plantation for 90 d, the disease index of each treatment was assessed for caculating the control efficiency of *B. cereus* strain S2 and Avermectin pesticide, respectively^42^.

## Additional Information

**How to cite this article**: Gao, H. *et al*. *Bacillus cereus* strain S2 shows high nematicidal activity against *Meloidogyne incognita* by producing sphingosine. *Sci. Rep.* **6**, 28756; doi: 10.1038/srep28756 (2016).

## Supplementary Material

Supplementary Information

## Figures and Tables

**Table 1:** Control effect of *B. cereus* S2 on *M. incognita* in pot experiment.

Treatments	Disease index	Control effect (%)
Water control	34.07 ± 3.0	−
*B. cereus* strain S2 culture	6.35 ± 1.52**	81.36 ± 6.72
*B. cereus* strain S2 supernatant	11.1 ± 3.92**	67.42 ± 2.61

^**^indicated very significant difference between treatment and control (*P *< 0.01).

**Table 2:** Growth characteristics of tomato.

Treatments	Percentage increase of growth indexes (mean ± standard deviation)					
	plant height	root length	plant fresh weight	plant dry weight	root fresh weight	root dry weight
*B. cereus* strain S2 culture	22.84 ± 6.61	27.16 ± 2.61	65.48 ± 5.37	94.63 ± 1.60	71.30 ± 0.96	220.73 ± 1.23
*B. cereus* strain S2 supernatant	25.79 ± 2.94	44.49 ± 1.23	96.03 ± 1.72	82.11 ± 0.60	31.78 ± 0.19	124.35 ± 0.68

**Table 3:** Control effect of *B. cereus* S2 on *M. incognita* in field experiment.

Treatments	Disease index	Control effect (%)
Water control	20.74 a	−
*B. cereus* strain S2 culture	8.51 ± 0.69 b	58.97
Avermectin	5.56 ± 1.23 c	73.19

Different lowercase letters indicated very significant difference between different treatments (*P* < 0.01).
