# Supplementary material for: Bacillus cereus strain S2 shows high nematicidal activity against Meloidogyne incognita by producing sphingosine
Source: Sci Rep. 2016 Jun 24;6:28756. doi: 10.1038/srep28756 (PMC4919635; doi:10.1038/srep28756)
Supplement: Supplementary Information [file srep28756-s1.doc]

***Bacillus cereus* strain S2 showed high nematicidal activity against *Meloidogyne incognita* by producing** **sphingosine**

Huijuan Gao, Gaofu Qi, Rong Yin, Hongchun Zhang, Chenggang Li, Xiuyun Zhao*

**Figure S1**

**a**

**

**

**

**

**

*

*

**b**

b

b

a

**Fig.S1.** **Nematicidal activity *B. cereus* strain S2 supernatants at different conditions.** a. Nematicidal activity of *B. cereus* supernatants between pH 2 and 12. Control: M9 buffer; S2, *B. cereus* strain S2 supernatants. 2-12: supernatant of *B. cereus* strain S2 culture was adjusted to pH 2, 4, 6, 8, 10 and 12, then nematicidal activity of supernatant was detected. * indicate the treatment groups are significant (*P* < 0.05) difference from the control. ** indicate the treatment groups are very significant (*P* < 0.01) difference from the control; b. Culture filtrate of *B. cereus*was treated with protease and detected nematicidal activity on *C.elegans*. E, culture filtrate of *B. cereus*was treated with protease. S2, culture filtrate of *B. cereus.*

**Figure S2**

**a**


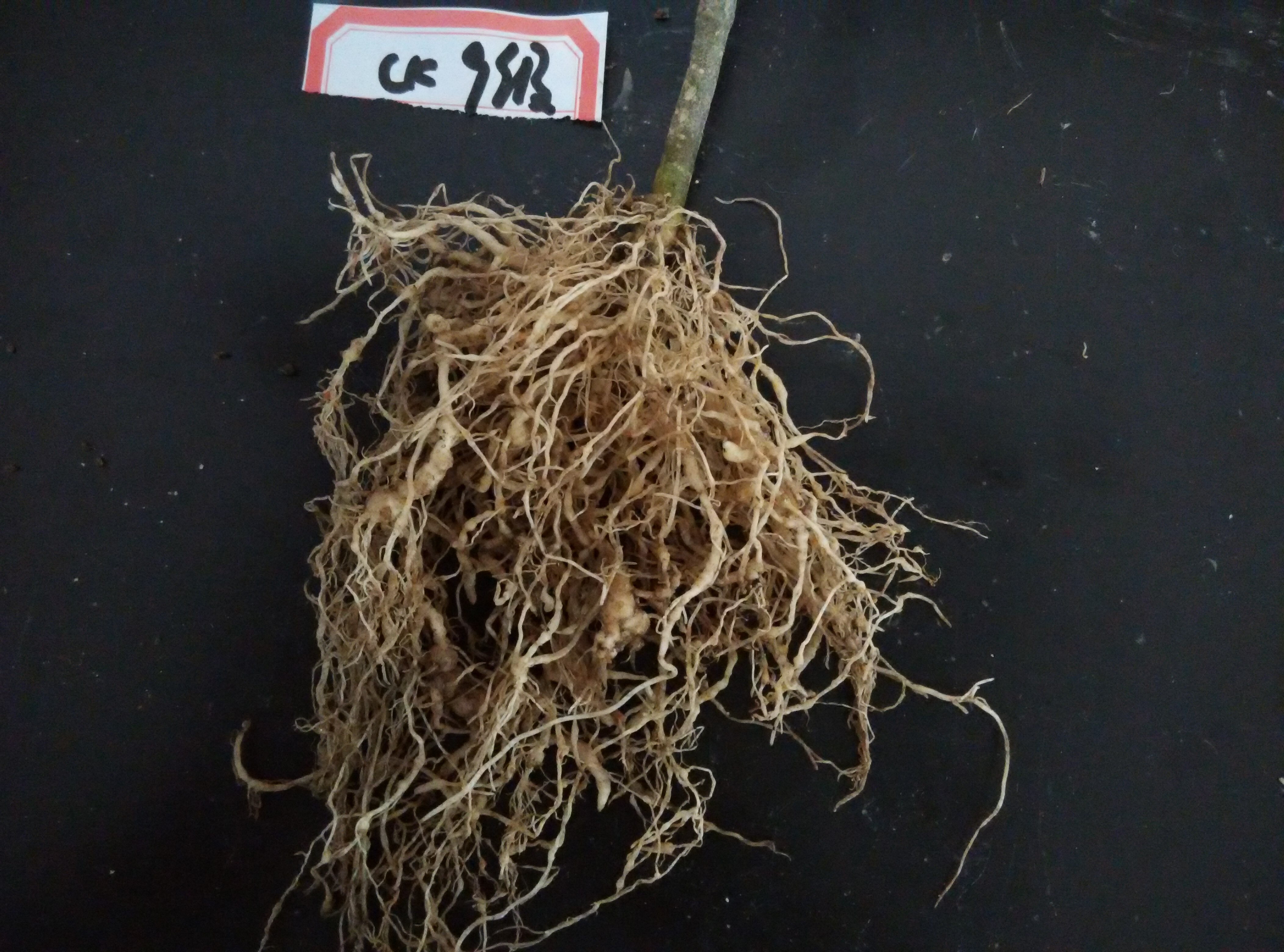

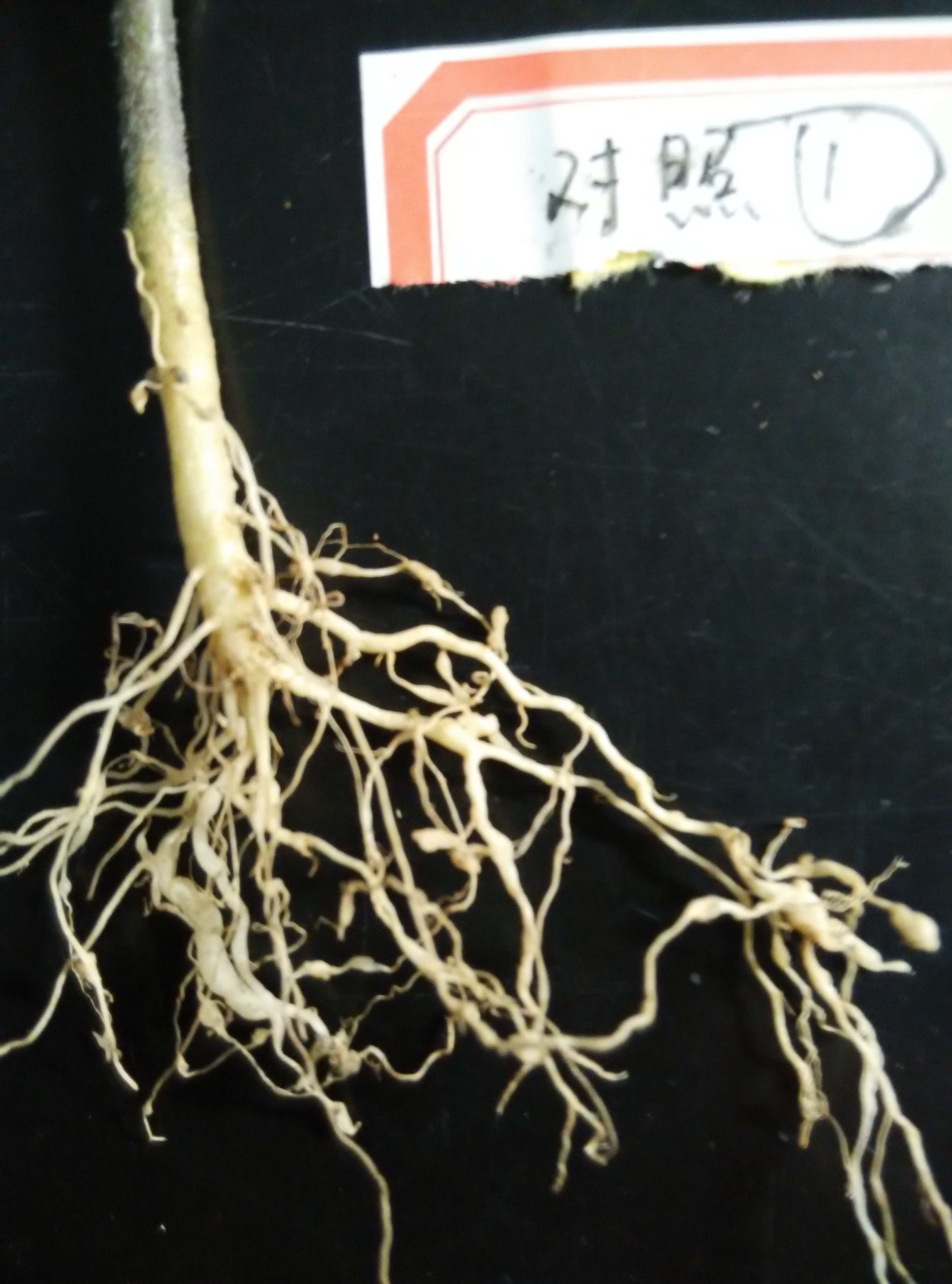


**b**


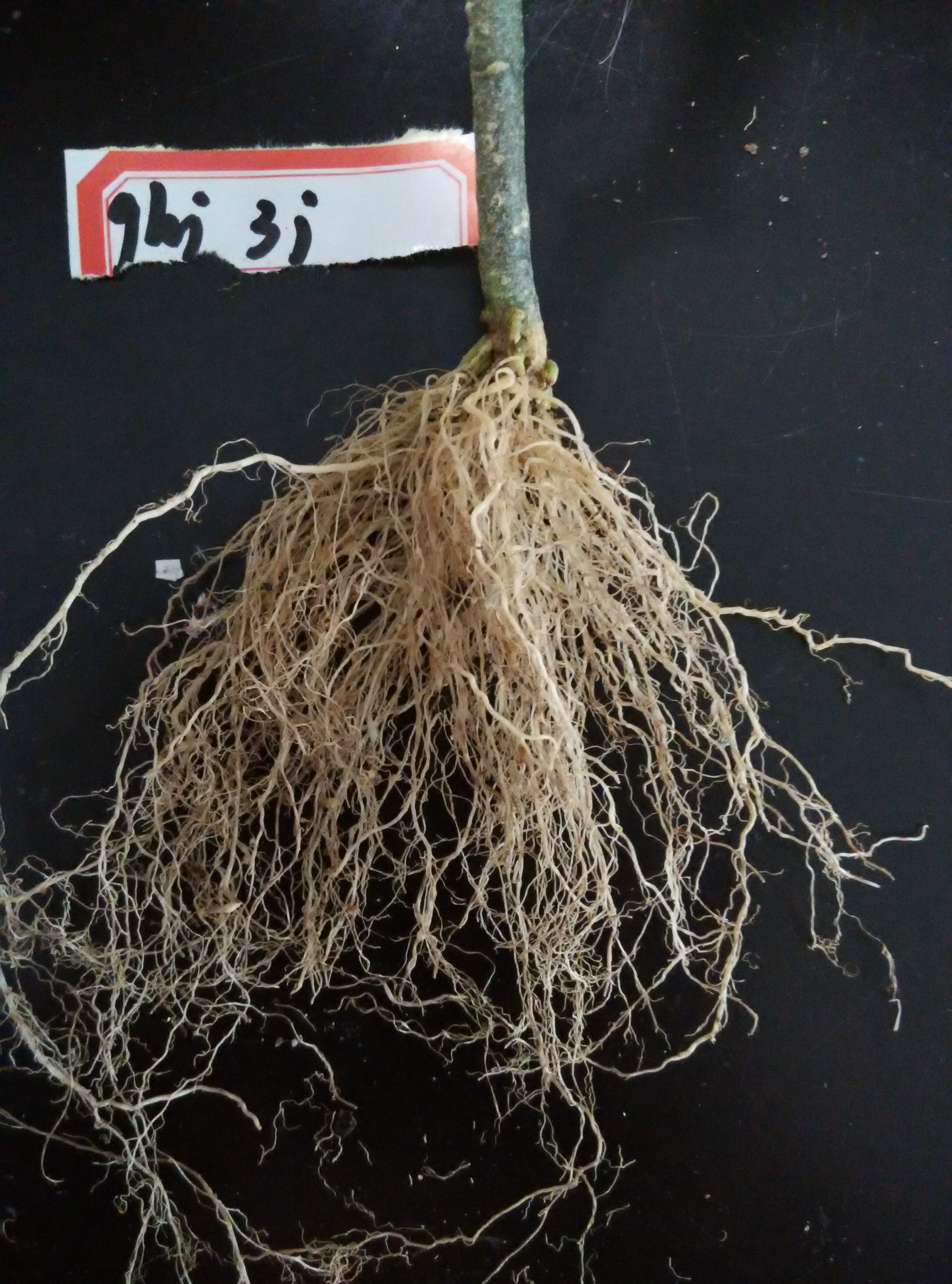

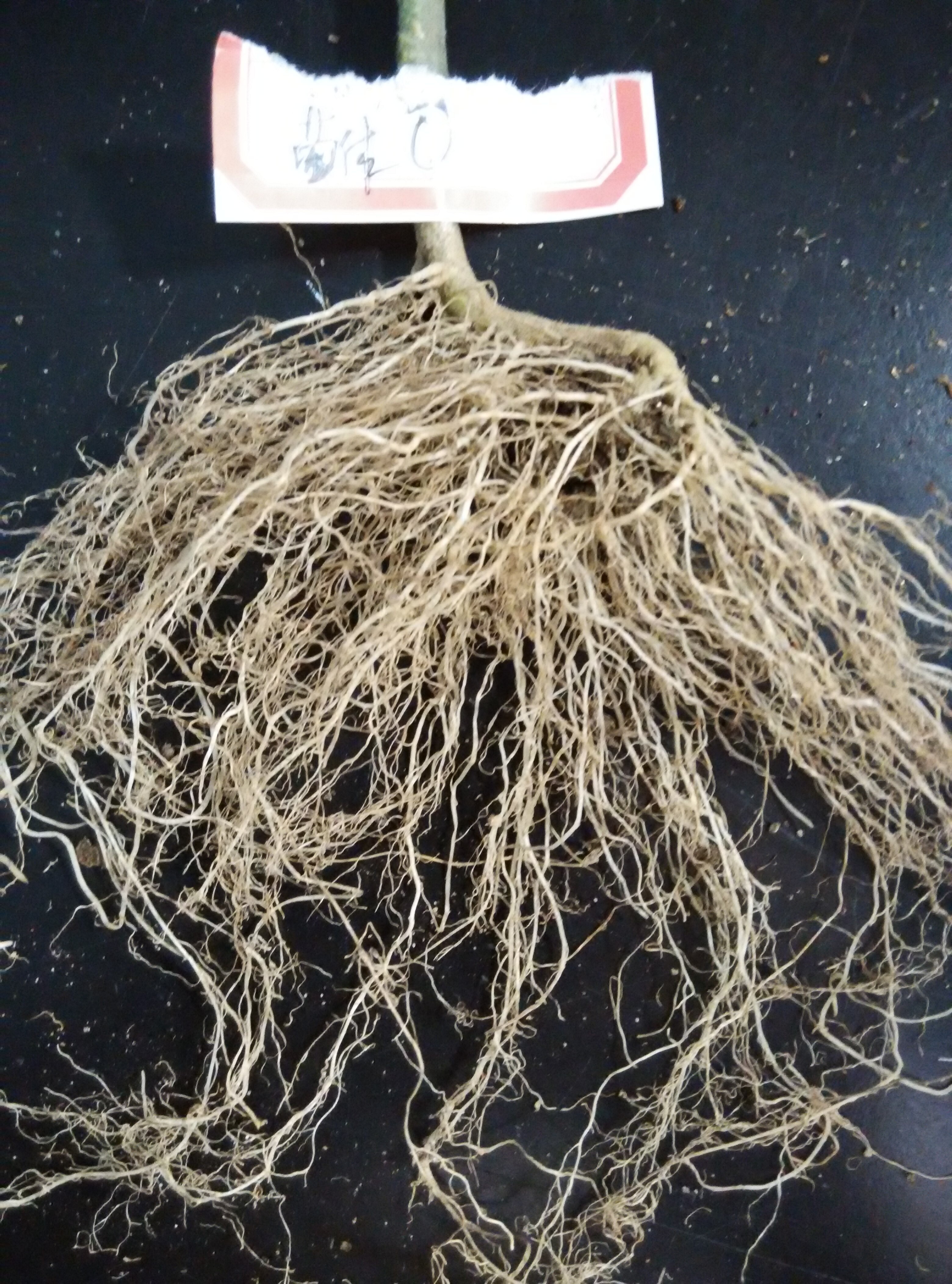


**c**


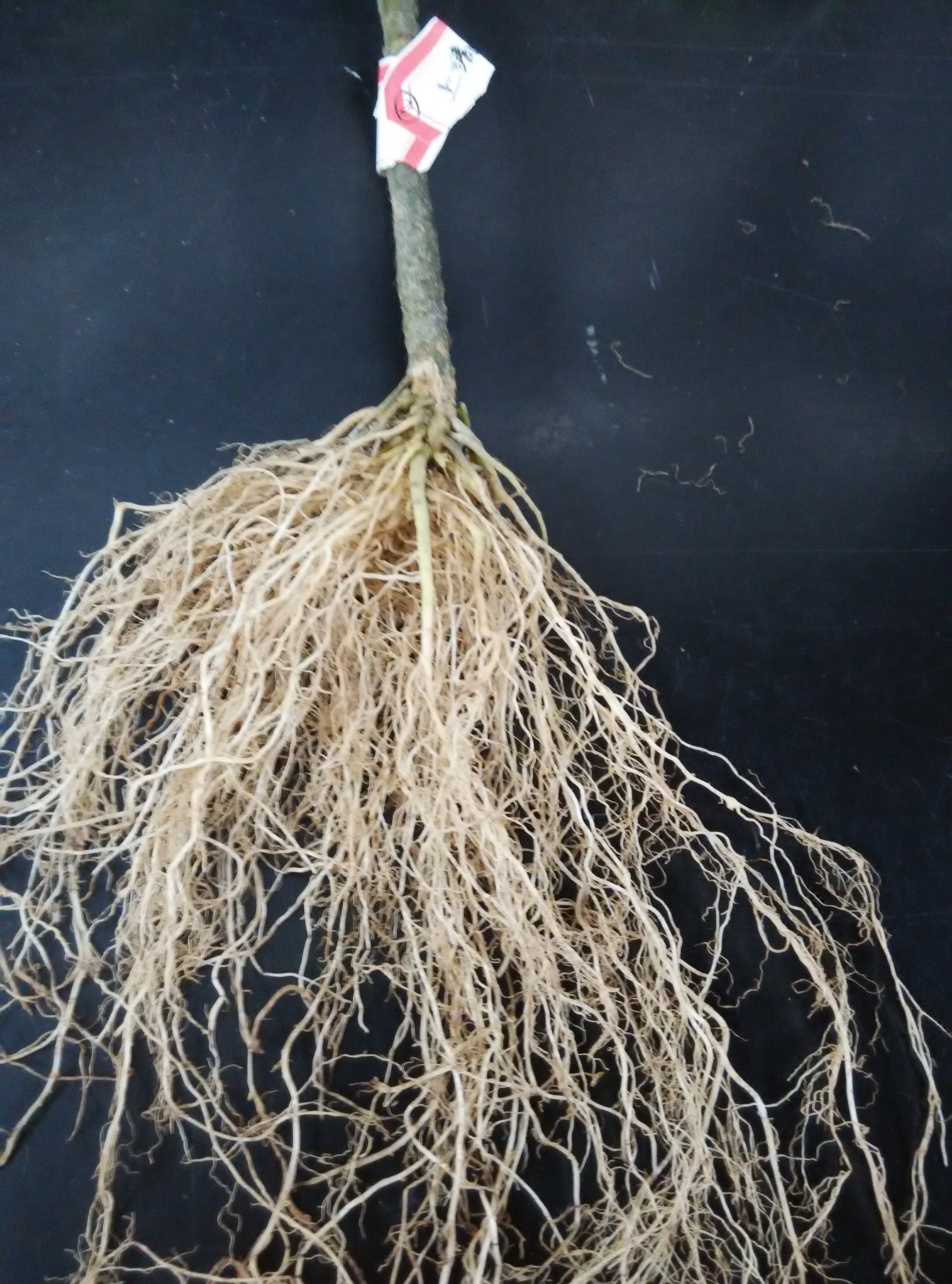

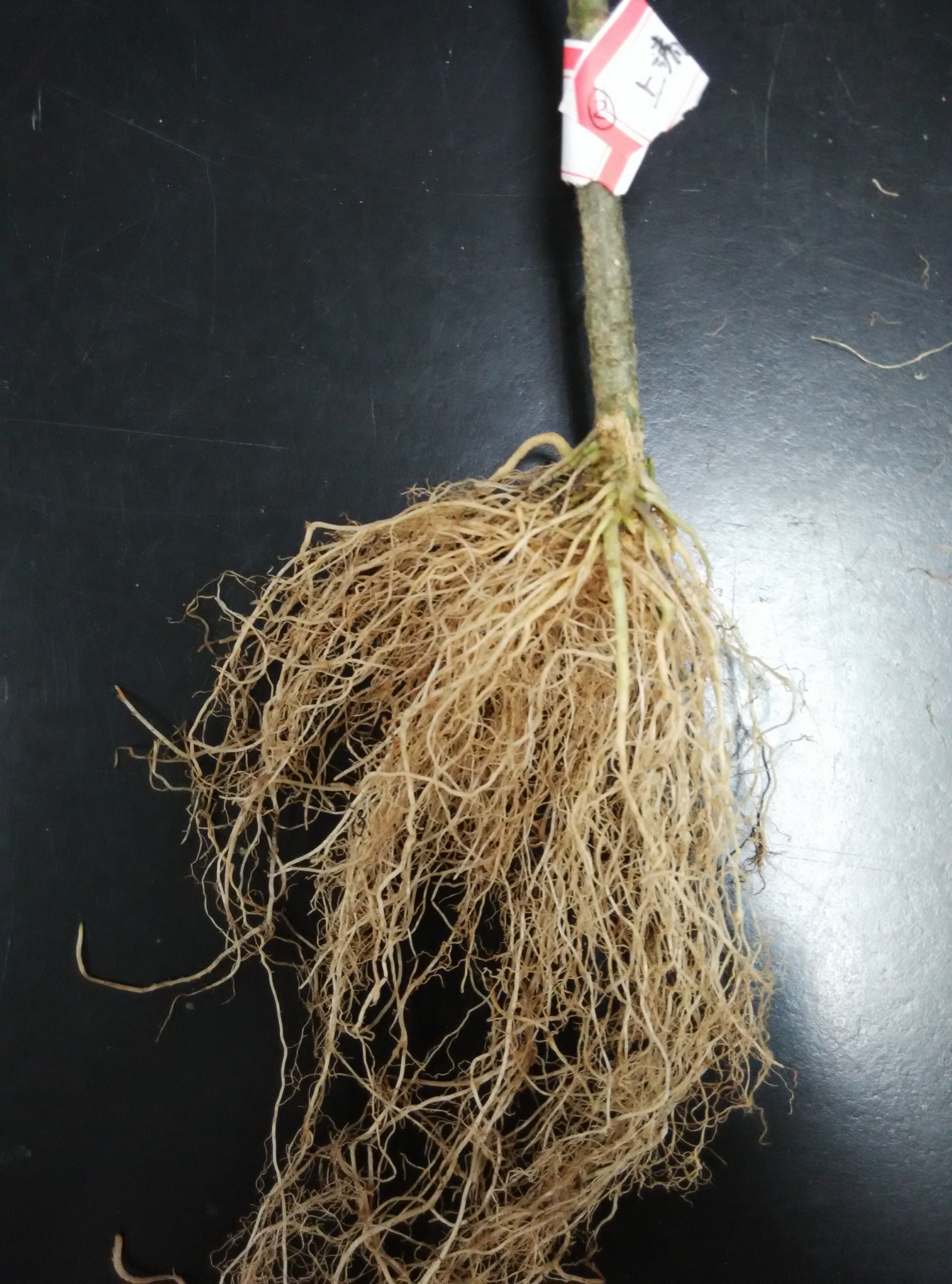


**Fig.S2. Root galling in tomato infested *M. incognita.*** a. Control plants irrigating water; b. Tomato inoculating *B. cereus* culture; c. Tomato inoculating *B. cereus* supernatant.
